# Benchmarking of five NGS mapping tools for the reference alignment of bacterial outer membrane vesicles-associated small RNAs

**DOI:** 10.3389/fmicb.2024.1401985

**Published:** 2024-07-19

**Authors:** Bojana Banović Đeri, Sofija Nešić, Ivan Vićić, Jelena Samardžić, Dragana Nikolić

**Affiliations:** ^1^Group for Plant Molecular Biology, Institute of Molecular Genetics and Genetic Engineering, University of Belgrade, Belgrade, Serbia; ^2^Department of Food Hygiene and Technology, Faculty of Veterinary Medicine, University of Belgrade, Belgrade, Serbia

**Keywords:** small RNAs, bacterial outer membrane vesicles, aligners, benchmarking, biotype

## Abstract

Advances in small RNAs (sRNAs)-related studies have posed a challenge for NGS-related bioinformatics, especially regarding the correct mapping of sRNAs. Depending on the algorithms and scoring matrices on which they are based, aligners are influenced by the characteristics of the dataset and the reference genome. These influences have been studied mainly in eukaryotes and to some extent in prokaryotes. However, in bacteria, the selection of aligners depending on sRNA-seq data associated with outer membrane vesicles (OMVs) and the features of the corresponding bacterial reference genome has not yet been investigated. We selected five aligners: BBmap, Bowtie2, BWA, Minimap2 and Segemehl, known for their generally good performance, to test them in mapping OMV-associated sRNAs from *Aliivibrio fischeri* to the bacterial reference genome. Significant differences in the performance of the five aligners were observed, resulting in differential recognition of OMV-associated sRNA biotypes in *A. fischeri*. Our results suggest that aligner(s) should not be arbitrarily selected for this task, which is often done, as this can be detrimental to the biological interpretation of NGS analysis results. Since each aligner has specific advantages and disadvantages, these need to be considered depending on the characteristics of the input OMV sRNAs dataset and the corresponding bacterial reference genome to improve the detection of existing, biologically important OMV sRNAs. Until we learn more about these dependencies, we recommend using at least two, preferably three, aligners that have good metrics for the given dataset/bacterial reference genome. The overlapping results should be considered trustworthy, yet their differences should not be dismissed lightly, but treated carefully in order not to overlook any biologically important OMV sRNA. This can be achieved by applying the intersect-then-combine approach. For the mapping of OMV-associated sRNAs of *A. fischeri* to the reference genome organized into two circular chromosomes and one circular plasmid, containing copies of sequences with rRNA- and tRNA-related features and no copies of sequences with protein-encoding features, if the aligners are used with their default parameters, we advise avoiding Segemehl, and recommend using the intersect-then-combine approach with BBmap, BWA and Minimap2 to improve the potential for discovery of biologically important OMV-associated sRNAs.

## 1 Introduction

The advancement of high-throughput sequencing technology has led to a burst of knowledge about the complexity and diversity of small RNAs (sRNAs), but has also raised new and specific bioinformatics challenges related to the analysis of sRNA-seq data (Baldrich et al., 2019; Diallo and Provost, 2020; Bezuglov et al., 2023). Most of these challenges are related to the short length of different sRNAs. One of them is ability/reliability of distinguishing functional sRNAs synthesized by the cell from the degradation products released during Next Generation Sequencing (NGS) sample preparation. Although the loss of very small RNAs (vsRNAs of approximately 8–15 nt) (Diallo et al., 2022) could be prevented by trimming only the specific adapter sequences used in sRNA-seq library preparation, correct mapping still remains an arduous task. Due to the short length of the reads, the frequent presence of different sequence variations of biological origin (as a result of post-transcriptional modifications and RNA editing of sRNAs) and the fact that some sRNAs are derived from repetitive regions, the main problems in mapping are ambiguous mapping (multi-mapping, cross-mapping) and/or the lack of mapping of some sRNAs. Furthermore, the expression values of the sRNAs obtained from the NGS data may not accurately reflect their absolute expression levels. This is due to the observed bias that depends on the use of different adapters, barcodes and the presence of complex RNA structures and modifications, all of which affect the efficiency of cDNA synthesis, while the GC-content of the different specific sRNAs’ affects the efficiency of PCR amplification (Raabe et al., 2014).

In this paper we focus exclusively on the correct mapping of bacterial sRNAs packaged in outer membrane vesicles (OMVs) to the bacterial reference genome and on testing different aligners to determine origin and biotype of bacterial sRNAs in OMVs. OMVs are extracellular vesicles (EVs) secreted by Gram-negative bacteria. Like eukaryotic EVs, OMVs are naturally secreted spherical nanoparticles containing all kinds of biomolecules including proteins, DNA and RNA, coated by a lipid bilayer (Caruana and Walper, 2020). Their cargo does not appear to be randomly packed cellular content, rather it is the result of a selection. This selected content is protected inside the EVs and safely delivered into the target cell, enabling efficient and specific intercellular communication. The interaction between cells mediated by EVs is considered an important, although not yet sufficiently explored means both for intraorganismic cellular communication and communication between organisms belonging to different species and even different kingdoms. OMVs are also considered to have great potential for biotechnological and biomedical applications (Sartorio et al., 2021).

Bacterial sRNAs are known to be 8 to 200 nucleotides long RNAs that originate from the bacterial chromosome, plasmids or phages and influence the transcriptional and post-transcriptional regulation of bacterial and/or host gene expression (Bloch et al., 2017; Barik and Das, 2018; Sousa et al., 2023). The different biotypes of bacterial sRNAs differ in structure, mechanism of action and degree of regulation, but can be broadly categorized into the following groups: trans-acting sRNAs (bind to target messenger RNA (mRNAs)) and cis-encoded sRNAs (bind to mRNAs, proteins, and DNA) (Brantl and Müller, 2021; Felden and Augagneur, 2021). Like other sRNAs, they can often have different modifications at their 3′ and 5′ ends or the ones placed inside (Felden and Gilot, 2018). Some of these bacterial sRNAs are packaged as OMV cargo depending on the growth phase and environmental conditions, enabling various interactions within and between kingdoms, some of which are well documented, while others remain to be studied (Ahmadi Badi et al., 2020). There is growing evidence that bacterial vesicles deliver their RNA into eukaryotic cells, affect host gene expression and induce phenotypic changes (Dauros-Singorenko et al., 2018). Thus, knowledge of the properties of OMV-associated sRNAs would be valuable in our attempt to understand the mechanism underlying these forms of host-bacteria communication and to harness the basic principles for biotechnological applications. Conclusions on how to improve the correct aligning and biotype determination of bacterial OMV sRNAs are an important step toward achieving this goal.

To date, bacterial OMVs have been found to contain large amounts of differentially packaged heterogeneous sRNAs of the following biotypes (discovered so far): mRNA-derived, tRNA-derived, rRNA-derived, pseudogene-derived sRNAs and ‘other’ class sRNAs. mRNA-derived sRNAs are derivatives of different regions of mRNAs (3′ and 5′ ends and coding regions) that are transcribed from the regions overlapping a 3′UTR/5’UTR or are cleaved from the parental mRNA by ribonucleases (RNases) or are transcribed independently and later processed by RNases (Iosub et al., 2021). Their functions are numerous and varied, i.e., in the utilization of an alternative carbon source, as a response to oxidative stress, to balance bacteriochlorophyll-based photosynthesis, etc. (Iosub et al., 2021; Ponath et al., 2022). The other major biotype is tRNA-derived sRNA as thermodynamically stable derivative of a polycistronic transport RNA (tRNA) precursor transcript or mature tRNA, which include three major types: (1) sequences of the external and internal transcribed spacer (ETS, ITS), (2) 5′ and 3’ tRNA halves, and (3) 5′ and 3’ tRNA-derived fragments (5’ tRFs and 3’ tRFs) (Li and Stanton, 2021). They are very abundant, which is consistent with the fact that tRNAs are the RNA species with the largest number of molecules in both eukaryotes and prokaryotes (Koeppen et al., 2016). Their functions are not yet fully characterized, but are thought to be diverse (e.g., one of many in modulating host nodulation (Ren et al., 2019)), as there are reports of various mRNAs predicted to be targets of tRNA-derived sRNAs (Diallo et al., 2022). The next important biotype is rRNA-derived sRNA, originating from ribosomal RNA (rRNA) like dodecaRNA (doRNA) with 12 core nucleotides and its 13-nucleotide variant C-doRNA (with 5′ cytosine). Pseudogene-derived sRNAs appear to be antisense RNAs derived from pseudogenes with different regulatory functions (Goodhead et al., 2020). sRNAs labeled ‘other’ include all sRNAs that do not fit into the previous four biotypes, such as transfer messenger RNAs (tmRNA), small bacterial RNAs with structural and functional similarities to both tRNA and messenger RNA (Zwieb et al., 1999) and several others. Overall, sRNA biotypes are highly diverse and vary within and between bacterial species and depend on the phase of the life cycle, environmental conditions and interactions with other organisms. Considering their different and specific functions, it is very important that no biotype is lost during OMV-associated sRNA-seq analysis.

Thus, the aim of this study was to test different and widely used NGS mapping tools for their performance in correctly aligning bacterial OMV sRNA-seq reads to the bacterial reference genome and their impact on sRNA biotype determination. Five NGS mapping tools selected for testing in this study were all used with their default parameters for pair-end reads and are listed below in alphabetical order:

1. *BBmap* is a global aligner written in the Java programming language based on the Smith–Waterman algorithm that uses sliding-window indexing with short kmers, without size or scaffold count limit and uses Simple Score (Levenshtein distance model) for mismatch evaluation (Bushnell et al., 2017). It looks for the alignment with the highest score, taking into account all bases in a sequence, but is very tolerant of errors and indels. The default parameters are numerous and include: kmer length = 13, break up fasta reads longer than 500, do not look for introns longer than 16,000, approximate minimum alignment identity to look for = 0.76, the minimum number of seed hits for candidate sites is 1, in case of ambiguity the first best site is used, pairing of reads without correct strand orientation is forbidden, initial average distance between paired reads is 100 and the maximum allowed distance between paired reads is 32,000, no attempt is made to try to rescue paired reads if the average insert size is greater than 1,200, the maximum allowed mismatches in the rescued read is 32, the band width is 0, do not analyze more than 800 alignments per read, discard frequently occurring kmers with low information, use quality scores when determining which read kmers to use as seeds, do not map reads with an average quality below 0, etc.
2. *Bowtie2* is a global aligner written in the C++ programming language that uses the Burrows-Wheleer-Transform (BWT) algorithm with modified Ferragina and Manzini (FM) matching algorithm and Substitutional Matrices score for mismatch assessment (Langmead et al., 2009; Langmead and Salzberg, 2012). It searches for unique, valid concordant and discordant alignments for each read. When it finds a valid alignment, it continues to search for alignments that are nearly as good or better until it has exceeded a limit on the search effort or has collected all the information needed to report an alignment. When Bowtie2 encounters a set of equally good alignments, it uses a pseudo-random number for selection and considers overlapping and consistency with concordant alignment. If Bowtie2 cannot find a paired-end alignment for a pair-end reads it looks for unpaired alignments for the constituent mates. For an alignment to be considered valid by Bowtie2, it must have an alignment score equal to or higher than the minimum score threshold. The default value for the minimum score threshold is −0.6 + −0.6 * L, where L is the read length. The other default parameters are: number of mismatches is 0, seed length is 20, seed used as a pseudo-random generator is 0, adding columns to allow gaps to solve dynamic programming problems is 15, disallow gaps within a certain number of positions at the beginning/end of the read is 4, no-unpaired alignments is false, no discordant alignments is false, no forward orientation is false, no reverse complement orientation is false, no overlapping mates is false, no mates that contain each other is false and mismatched bases at a high-quality position in the read are penalized.
3. *BWA* is an aligner written in the C++ programming language that uses BWT with an FM matching algorithm to find short matches so-called seeds and Simple Score (Levenshtein distance model) for the evaluation of mismatches (Li and Durbin, 2009). The BWA-MEM algorithm is based on the seeding alignments with maximal exact matches (MEMs) and extending seeds with the affine-gap Smith-Waterman algorithm (SW). BWA-MEM performs local alignment, using soft clipping for primary alignment and hard clipping for supplementary alignments. If the best match is not very repetitive, it searches for all matches that contain another mismatch, otherwise it only finds all equally good matches. For this reason, BWA-MEM can generate multiple primary alignments for different parts of a query sequence, while the quality of the bases is not considered when evaluating the matches. In paired-end mode, it pairs all hits found and performs a Smith-Waterman alignment for unmapped reads to rescue reads with a high error rate and to fix possible alignment errors in high-quality anomalous pairs. In paired-end mode, the mem command will infer the read orientation and the insert size distribution from a batch of reads. The default parameters include: minimum seed length is 19, internal seed length is 1.5, skip seed threshold is 10,000, band width is 100, dropoff is 100, drop chain threshold is 0.5, rounds of made rescues is 100, skip mate rescue is false, skip pairing is false, score threshold is 30, as well as a number of penalties for mismatch, gap, clipping and unpairing.
4. *Minimap2* is a global aligner written in the C programming language based on the BWT algorithm (uses hashed minimizers in the reference genome and query sequences as seeds to find matches as seed-chain-aligner) and Substitutional Matrices Score for mismatch evaluation (Li, 2018). For each query sequence, it takes query minimizers as seeds, finds exact matches called anchors and identifies sets of colinear anchors as chains by applying dynamic programming to expand from the ends of the chains and regions between adjacent anchors in the chains. To adapt Minimap2 to short reads and to allow comparison with other aligners, we had to add two recommended options: ‘-a’ to generate output alignments in SAM format (instead of Minimap2’s default output PAF) and ‘-x sr’ to preset multiple parameters with suitable defaults for mapping short reads against reference genome. Minimap2 default parameters include: kmer size of 15, minimizer window size of 10, split index for each 8G input bases, filtering out the top 0.0002 fraction of repetitive minimizers, stopping chain elongation if there are no minimizers in 5000 bp, maximum intron length is 200,000, maximum fragment length is 800, minimum number of minimizers on a chain is 3, minimum chaining score is 40, minimum secondary-to-primary score ratio is 0.8, retaining at most 5 secondary alignments, matching score is 2 and multiple mismatch/gap penalties.
5. *Segemehl* is a local aligner written in the C programming language based on the BWT algorithm (it uses enhanced suffix arrays to find the seeds with the best results) and uses Substitutional Matrices Score for mismatch evaluation (Hoffmann et al., 2009). Seeds that Segemehl finds can contain insertions, deletions and mismatches (differences). The number of allowed differences within a single seed is crucial for the runtime of the program. The default parameters include: detect splits/spliced reads = none, accuracy is 90, dropoff is 8, search seeds with difference of 1 and jump size 0, max E-value is 5, minimum length of queries is 12, minimum length of a spliced fragment is 20, minimum coverage for spliced transcripts is 80, minimum score of a spliced fragment is 18, report spliced alignment with score 0.9 only if it is larger than the next best spliced alignment, maximum size of pair-end inserts in case of multiple reads is 200,000, query seed is skipped if it matches more than 100 times, it penalizes mismatch during extension and it reports only the best hits by default.

Each of these aligners performs differently, depending on the alignment algorithm and method of mismatch evaluation. Although they all aim to align reads as accurately as possible against a reference genome, they differ, e.g., in their accuracy, computational time, depending on the characteristics of the NGS data and the reference genome used in the analysis. While the computational time depends on the efficiency of the algorithm and the characteristics of the hardware used for its execution, the accuracy of the aligner is indirectly estimated based on the expected complete alignment of the reads to the reference genome. Therefore, the accuracy can be estimated using various parameters, e.g., the alignment and assignment rates, the percentage of unmapped/multimapped reads, coverage, etc. The alignment rate indicates the percentage of the total number of reads that have been aligned to the reference genome and the assignment rate indicates the percentage of aligned reads that have been assigned to a position in the reference genome. The average number of reads covering a specific region (e.g., gene or chromosome) or the entire reference genome represents the gene/chromosome/overall coverage and provides a further indication of whether or not there is a potential error in the alignment of the reads. If the aligner cannot determine which region of the reference genome is the correct match for the read, it leaves this read unmapped (matching region not found) or marks it as multimapped (match at multiple positions). If the reference genome is complete, a large number of unaligned reads can sometimes indicate a low accuracy of the aligner (Musich et al., 2021). However, this also depends on the reads (i.e., spliced RNA-seq reads mapped to the DNA reference genome, sRNA-seq reads containing biologically post-transcriptionally edited sequences leading to mismatches, etc.) and the complexity of the reference genome (number of repetitive sequences, duplicated regions, homopolymeric regions, hotspots for polymorphisms, etc.). Paired-end reads can sometimes be helpful to resolve repetitive parts of the reference genome, but only if a read at either end comes from a non-repetitive part of the reference genome and can be correctly assigned. In addition, each aligner assigns a mapping quality (MAPQ) score to each read, which indicates the probability that a read is misaligned. It is important to consider the overall performance of each aligner with respect to the characteristics of the specific NGS dataset and the reference genome. Selecting the most appropriate aligner(s) for a particular NGS analysis ensures the best possible biological interpretation of the results.

In this study, we selected a broad length range sRNAs-seq data from *Aliivibrio fischeri* OMVs, available in the National Center for Biotechnology Information (NCBI) database, to evaluate and compare the performance of five aligners. *A. fischeri* is a Gram negative marine bacterium known for its bioluminescent symbiosis with the squid *Euprymna scolopes,* in which the *A. fischeri* sRNA SsrA (tmRNA) and the associated chaperone Hfq (a ubiquitous, Sm-like RNA binding protein) play an essential role (Moriano-Gutierrez et al., 2020; Tepavčević et al., 2022). The performance of the five mapping tools was evaluated in terms of alignment and assignment rates, percentage of unmapped/multimapped reads, computational time and additionally the number of different biotypes assigned to sRNAs by each aligner on this particular dataset. The results obtained are discussed in terms of the potential of the tested tools to detect all (or the majority) of the biologically important OMV sRNAs represented in the analyzed NGS dataset.

## 2 Materials and methods

### 2.1 Dataset

The *Aliivibrio fischeri* OMV RNAseq dataset was downloaded from the National Center for Biotechnology Information Sequencing Read Archive (NCBI SRA) database – accession number PRJNA629425^1^ and used for comparison of tools. This dataset consists of six samples of OMV-associated sRNA-seq reads (two sets of triplicates of RNA-seq data from OMVs produced by *A. fischeri* wild type strain ES114 and mutant-derived strain SMG7 lacking tmRNA (SsrA), obtained by Illumina HiSeq 2,500 sequencing of paired-end stranded RNA libraries preselected for <300 nucleotide size) and contains sRNAs of different lengths, varying from 10 to 76 nucleotides. The size of the samples was 131.3 Mb, 152.3 Mb and 132.3 Mb for triplicates of the wild type strain and 140.4 Mb, 153.2 Mb and 157.1 Mb for triplicates of the mutant strain.

The reference genome and annotation file of *A. fischeri* wild type strain ES114 were downloaded from the NCBI SRA database with accession number PRJNA12986.^2^ The reference genome consists of two chromosomes and one plasmid: chromosome I with a total length of 2,897,536 bp, chromosome II with a total length of 1,330,333 bp and plasmid pES100 with a total length of 45,849 bp. The annotation file shows that the *A. fischeri* wild type strain ES114 genome encodes the following RNA biotypes: mRNA (3818), rRNA (37), tRNA (118), pseudogenes (5) and ‘other’ (10, including one tmRNA). Of the rRNAs copies the *A. fischeri* genome contains 13 copies of 5S (12 on chromosome I, one on chromosome II), 12 copies of 16S (11 on chromosome I, one on chromosome II) and 12 copies of 23S (11 on chromosome I, one on chromosome II). Each tRNA in the *A.fischeri* genome was also present in certain number of copies, namely: tRNA-Trp (3), tRNA-Glu (6), tRNA-Ile (3), tRNA-Ala (5), tRNA-Asp (7), tRNA-Leu (11), tRNA-Asn (7), tRNA-Phe (4), tRNA-Thr (8), tRNA-Ser (8), tRNA-Arg (9), tRNA-Gln (5), tRNA-Met (7), tRNA-Pro (3), tRNA-Val (6), tRNA-Gly (11), tRNA-Cys (3), tRNA-Tyr (6), tRNA-Lys (4) and tRNA-His (2). Protein-encoding genes (3818) were present without copies on both chromosomes (2,586 on chromosome I and 1,175 on chromosome II) and on the plasmid (57). In addition, five pseudogenes (two on chromosome I and three on chromosome II) and ten features of the class ‘other’ (nine on chromosome I, including one tmRNA and one on chromosome II) were present in the *A. fischeri* genome without copies.

### 2.2 Comparison of the tools

**Figure 1:**
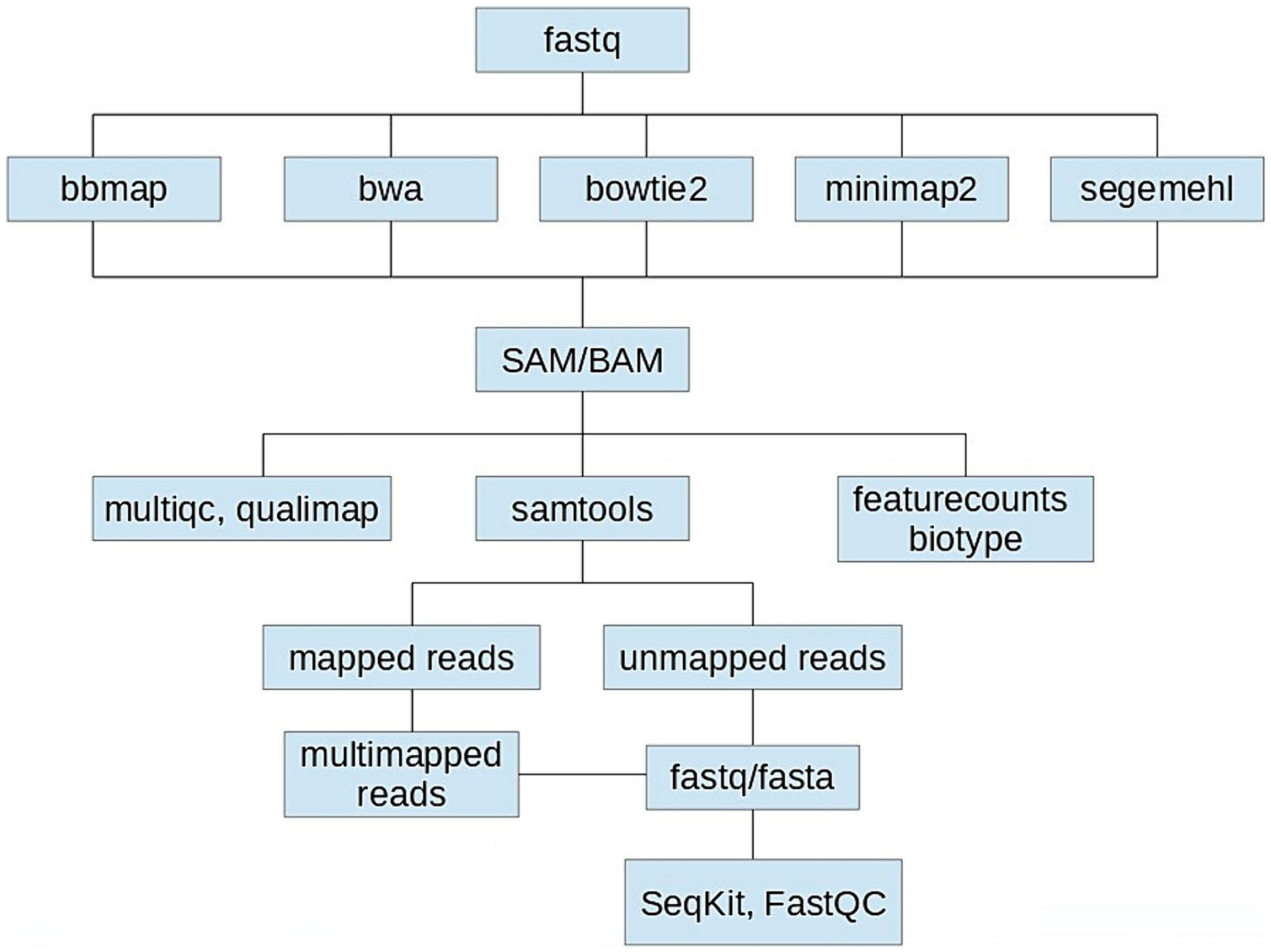
Schematics of the performance comparison of five aligners: BBmap (v39.01), Bowtie2 (v2.3.4.1), BWA (v0.7.17), Minimap2 (2.24), and Segemehl (v0.3.4) using their default settings when mapping the *Aliivibrio fischeri* OMV-associated sRNAseq dataset to its reference genome.

Using a computer configuration based on AMD Ryzen 72,700 U with Radeon Vega Mobile Gfx, 8 CPUs, 8GB DDR4 memory and 128 GB SSD + 1000GB HDD, we tested five aligners: BBmap (v39.01), Bowtie2 (v2.3.4.1), BWA (v0.7.17), Minimap2 (2.24) and Segemehl (v0.3.4) using their default settings (Figure 1).

The SAM files obtained were used to generate BAM files from which the aligned reads could be counted. The performances of the aligners were evaluated based on alignment rates (ratio of aligned and input reads), assignment rates (ratio of assigned and aligned reads), computational times and the total number and types of known sRNA biotypes detected. The alignment and assignment metrics used for the comparison were calculated with samtools (v1.3.1), multiqc (v.1.16) and qualimap (v2.2.2). The biotypes of the mapped reads were determined with featurecounts (v2.0.6) based on the annotation file of *A. fischeri* strain ES114.

In addition, for each aligner unmapped and multimapped reads were separated from the uniquely mapped reads, sorted and compared using samtools (v1.3.1) and seqkit (v.2.6.1) and the results were analyzed using FastQC (v.0.11.5).

Visual inspection of the mapped reads was performed using IGV-Linux-2.16.2.

### 2.3 Statistics

Prior to any statistical analysis, the normality of the data with continuous values was checked by applying the Shapiro-Wilks test (*p* > 0.05).

For the metrics, mapping quality, coverage, overall error rate, and indel types of five aligners, a general linear model was used, with Bonferroni correction for multiple *post hoc* comparisons. Because some results are presented in relative form and for model normalization, the total number of reads was included in the model for covariate adjustment. The results are presented as mean ± standard deviation. A probability level of *p* < 0.05 was used as the statistical significance threshold. Compact Letter Display (CLD) was used to display the results of pairwise comparisons between means, so that means with the same letter are not statistically significantly different from each other, while means with different letters are statistically significantly different at a specified statistical significance level.

The sensitivity (Sn) and specificity (Sp) between two aligners were calculated as the ratio of identified unique small RNAs and the number of aligner-specific small RNAs, respectively. The lower the specificity of the aligner, the higher the number of unique small RNAs identified by that aligner compared to another. Cohen’s Kappa was used to quantify the level of agreement between two aligners. Kappa values between 0.81 and 1.0 indicate an almost perfect agreement, while values below 0.20 indicate a low agreement. The Sn, Sp and Kappa values are presented as median with minimum and maximum values.

Statistical data analysis was performed using SPSS 21 (SPSS Inc., Chicago, IL, USA).

## 3 Results

The alignment and assignment rates and computational time were used to analyze how each aligner with its default parameters affects the mapping of *A. fischeri* OMV sRNA reads to the bacterial reference genome (Table 1). The highest alignment rate (for properly paired reads) was obtained with BBmap, followed by Segemehl, Bowtie2 and BWA (these two performed the same) and Minimap2, in descending order, but observed differences were only statistically supported for BBmap and Segemehl. The highest assignment rate was obtained for BBmap, followed by Bowtie2, BWA, Minimap2 and Segemehl, in descending order, but the observed differences were only statistically supported for BBmap and Segemehl. Segemehl had the lowest assignment rate compared to the other mapping tools, which is due to a very high percentage of multimapped reads. The computational time was shortest for BBmap and Minimap2, followed by BWA, then Bowtie2 and finally Segemehl, which required the longest computational time.

**Table 1:** Overview of the mean values of the metrics calculated over six ***A. fischeri*** OMV sRNAs samples for each of the five aligner tools tested, in alphabetic order.

Aligner	BBmap	Bowtie2	BWA	Minimap2	Segemehl
Mapped reads (%)	99.86 ± 0.01^a^	99.80 ± 0.01^a^	99.45 ± 0.04^b^	99.11 ± 0.08^c^	99.65 ± 0.02^d^
Mapped and paired reads (%)	99.73 ± 0.02^a^	99.64 ± 0.03^a^	98.93 ± 0.08^b^	98.29 ± 0.15^c^	99.29 ± 0.03^d^
Properly paired reads (%)	99.40 ± 0.04^a^	96.49 ± 0.67^b^	96.49 ± 0.57^b^	96.00 ± 0.61^b^	99.26 ± 0.04^a^
Assigned reads (%)	97.43 ± 0.42^a^	96.27 ± 0.57^b^	96.17 ± 0.52^b^	95.67 ± 0.55^b^	26.30 ± 0.13^c^
Computational time (min)	3.00	35.00	5.00	3.00	37.00

The results are presented as mean ± standard deviation. Different letters in the same row denote statistical significance at the *p* < 0.05 level.

Overall analysis of mean mapping quality (MAPQ), overall coverage and mean coverage per chromosome/plasmid (Table 2) showed that the highest MAPQ was recorded for BWA and Minimap2, followed by BBmap, Bowtie2 and Segemehl, in descending order, with statistical support for the observed differences. The highest overall coverage was recorded for BBmap, followed by Minimap2, Bowtie2, Segemehl and BWA, but without statistical significance. Examination of the mapping per chromosome/plasmid showed that the coverage of the first chromosome and plasmid was comparable between the mappers, while there were significant differences in the coverage of the second chromosome. While BBmap showed the highest coverage of the first chromosome, it had the lowest coverage of the second chromosome. Low coverage of the second chromosome was also observed in Segemehl, while it was statistically significantly higher in the other three aligners and close to the mean coverage.

**Table 2:** Overview of mean mapping quality (MAPQ), overall coverage, and mean coverage per chromosome/plasmid for five aligners in six *A. fischeri* OMV sRNAs samples.

Parameter	Chromosome	BBmap	Bowtie2	BWA	Minimap2	Segemehl
MAPQ	I, II, pES100	40.43 ± 0.35^a^	38.53 ± 0.31^b^	56.54 ± 0.38^c^	56.21 ± 0.39^c^	25.90 ± 0.27^d^
Coverage (X)	I, II, pES100	98.37 ± 7.38^a^	96.63 ± 7.31^a^	96.52 ± 7.30^a^	97.29 ± 2.98^a^	96.59 ± 7.31^a^
Mapped bases	I	419212428.67 ± 31320651.88^a^	378125009.50 ± 28670179.49^a^	378672927.17 ± 28705581.04^a^	377808067.67 ± 28630584.74^a^	403397590.67 ± 29544348.50^a^
	II	788589.50 ± 140962.12^a^	34417985.33 ± 2503393.24^b^	33421376.33 ± 2433981.21^b^	33549085.33 ± 2457007.83^b^	8972310.00 ± 1873628.12^c^
	pES100	419310.33 ± 110670.27^a^	419131.17 ± 110488.57^a^	418523.17 ± 110490.90^a^	417837.17 ± 110352.89^a^	418759.67 ± 110538.10^a^
Mean coverage	I	144.68 ± 10.81^a^	130.50 ± 9.89^a^	130.69 ± 9.91^a^	130.39 ± 9.88^a^	139.22 ± 10.20^a^
	II	0.59 ± 0.10^a^	25.87 ± 1.88^b^	25.12 ± 1.83^b^	25.21 ± 1.85^b^	6.74 ± 1.41^c^
	pES100	9.14 ± 2.41^a^	9.14 ± 2.41^a^	9.13 ± 2.41^a^	9.11 ± 2.41^a^	9.13 ± 2.41^a^

The results are presented as mean ± standard deviation. Different letters in the same row denote statistical significance at the *p* < 0.05 level. Chromosome I total length is 2,897,536 bp, chromosome II total length is 1,330,333 bp and plasmid pES100 total length is 45,849 bp.

The general error rates for all aligners were < 0.3% for Bowtie2, BWA, Minimap2 and Segemehl and < 2% for BBmap, which was statistically significantly higher than for other four aligners. The frequency of insertions was lower than that of deletions in all mappers, while homopolymeric indels were most frequent in all mappers. The highest insertion rate was observed in Segemehl and decreased in the following order: Bowtie2, followed by BBmap, BWA and Minimap2 (the last three performed the same), with statistical support for the observed differences. The statistically significant higher deletion rate was observed with Segemehl compared to other aligners and decreased with BBmap, Bowtie2, BWA and Minimap2 (the last two performed equally), but these other differences were not statistically supported. The homopolymer indels rate was the highest with BWA, followed by Minimap2, BBmap, Bowtie2 and Segemehl, with statistically supported differences for all aligner pairs except between BWA and Minimap2. These results are summarized in Table 3.

**Table 3:** Overview of the general error rate and indel types detected by five aligners in six *A. fischeri* OMV sRNAs samples.

Mismatch and indel type	BBmap	Bowtie2	BWA	Minimap2	Segemehl
General error rate	1.89 ± 0.56^a^	0.19 ± 0.01^b^	0.14 ± 0.01^b^	0.13 ± 0.01^b^	0.18 ± 0.01^b^
Mapped reads with insertion (%)	0.07 ± 0.001^a^	0.19 ± 0.04^b^	0.03 ± 0.004^a^	0.03 ± 0.004^a^	0.54 ± 0.06^c^
Mapped reads with deletion (%)	0.24 ± 0.02^a^	0.23 ± 0.03^a^	0.18 ± 0.03^a^	0.18 ± 0.03^a^	1.01 ± 0.07^b^
Homopolymer indels (%)	45.54 ± 1.58^a^	37.55 ± 3.02^b^	57.37 ± 1.37^c^	54.74 ± 5.53^c^	27.05 ± 3.42^d^

The results are presented as mean ± standard deviation. Different letters in the same row denote statistical significance at the *p* < 0.05 level.

The analysis of unmapped reads, which were present in all aligners at a generally low percentage (0.02–0.06%, from BBmap with the lowest to Minimap2 with the highest), showed that unmapped reads were present over the entire length range of the dataset. While some of them could not be mapped to the reference genome at all, others could be fully mapped at multiple locations in the genome and in different orientations. Their average GC ranged from 49 to 51%. Comparison of unmapped sequences between each pair of aligners showed that 35.8 to 100% of the sequences in six samples matched and contained a subset of 887 to 1,391 sequences that had not been mapped by all five aligners, as well as 27–863 overrepresented sequences in the six samples. In addition, two overrepresented kmers (‘TTTATTC’ and ‘TTTTATT’) were detected in unmapped reads of one sample by all five aligners.

All aligners showed a similarly high percentage of ambiguous/multimapped reads, approximately 96.5%, containing sequences over the entire length range of the dataset. These reads could be fully mapped at two or more sites in the genome in the same orientation. Comparison of ambiguous/multimapped reads between pairs of aligners showed a subset of 88.22 to 95.72% of multiply mapped sequences shared by all five aligners, with a mean GC of 51 to 52% and 141–159 overrepresented sequences and 20 different kmers across the six samples. Ambiguous/multimapped reads are considered further in the context of the biotypes to which they were assigned by each aligner.

**Figure 2:**
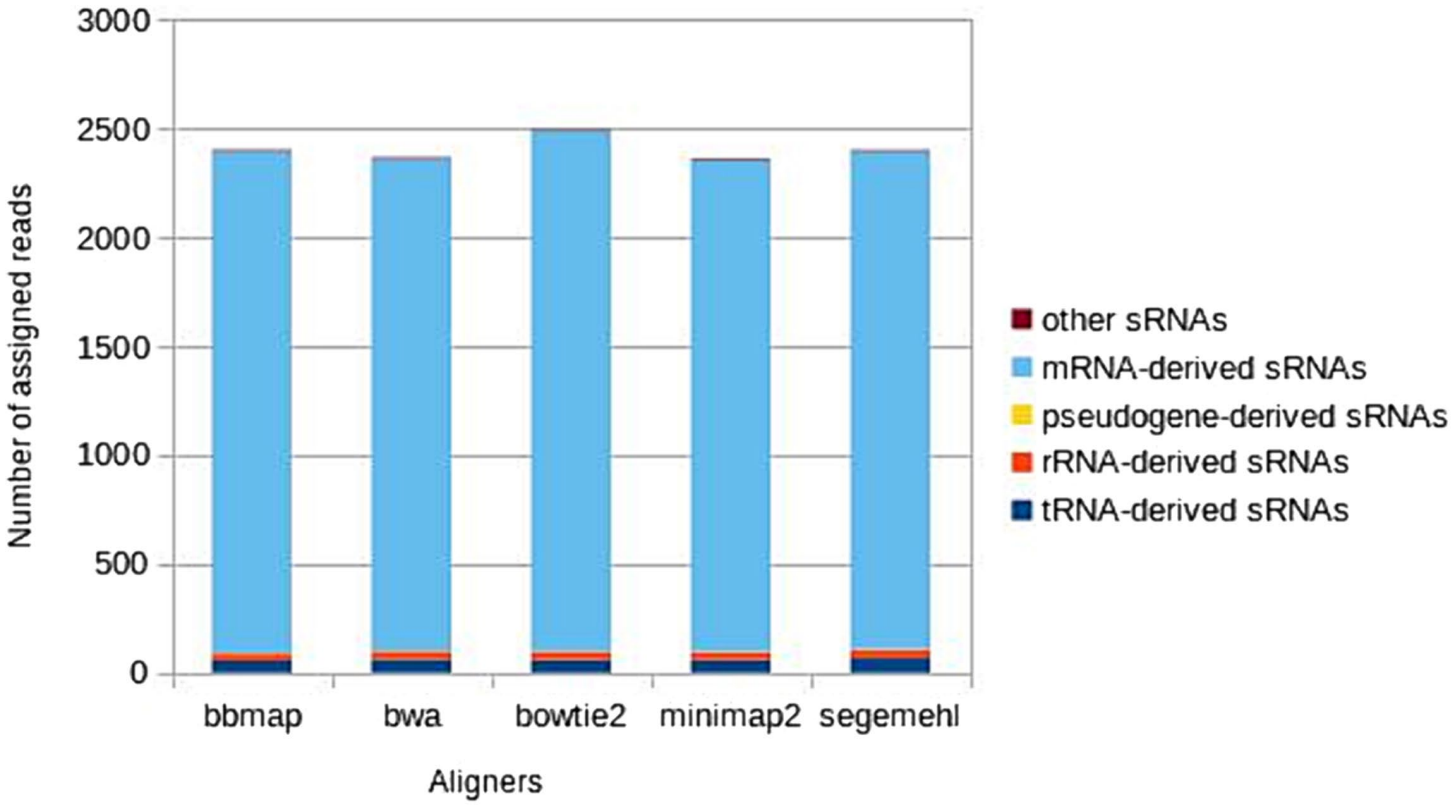
Distribution of the main sRNA biotypes detected by each aligner, calculated as the mean across six *A. fischeri* OMV sRNAs samples.

The five major biotypes (mRNA-, tRNA-, rRNA-, pseudogene-derived and ‘other’ sRNAs) represented in the *A. fischeri* annotation file were all recognized in the results of each aligner, but the number of unique representatives of each biotype that were recognized varied between aligners (Figure 2). Alignment most strongly influenced the recognition of biotypes associated with mRNA-derived, tRNA-derived and rRNA-derived sRNAs. The different aligners associated the reads differently with the specific sRNAs of each biotype, resulting in some sRNAs not being recognized by some aligners and the number of some reads associated with specific sRNAs differed between aligners. In contrast, when recognizing pseudogene-derived sRNAs and sRNAs from the ‘other’ class all aligners recognized the same ones, only the number of some reads associated with specific sRNAs of these two biotypes differed between the aligners.

Consistently across all six samples, most unique mRNA-derived sRNAs were assigned by Bowtie2, followed by Segemehl, BBmap, BWA and Minimap2 (in descending order). Most unique tRNA-derived sRNAs (in descending order) were assigned by Segemehl, BWA, Bowtie2, Minimap2 and BBmap (BWA and Bowtie2 as well as Minimap2 and BBmap were equal in their performance in one sample; additionally, in one of six samples the descending order was changed – again Segemehl detected the highest number of unique tRNAs and BBmap the lowest, but the three remaining aligners changed their order so that Segemehl was followed by Minimap2, BWA and then Bowtie2). In contrast, for rRNA-derived sRNAs Bowtie2, BWA, Minimap2 and Segemehl all assigned the reads to the same unique sRNAs (differing only in the number of reads assigned to each), while BBmap assigned the reads to a smaller number of unique rRNA-derived sRNAs than the other four mappers. The differences between the five aligners were consistent between samples (with a single exception in the tRNA-derived sRNAs of one sample), revealing the same preferences of each aligner in assigning reads to specific copies or genomic regions and affecting biotype recognition in the same way in six samples. All differences between the aligners in the assignment of sRNAs to mRNA-derived, tRNA-derived and rRNA-derived biotypes are listed in Supplementary Table S1.

The most important differences observed in the assignment of biotypes by the aligners tested are summarized below:

1. All aligners showed the greatest differences in the assignment of reads to unique protein-encoding features represented on two chromosomes and the plasmid (Table 4), along with differences in the number of reads assigned to each feature (Supplementary Table S1). When detecting mRNA-derived sRNAs, the specificity of the aligners decreased, and the sensitivity increased in the order Minimap2-BWA-BBmap-Segemehl-Bowtie2. The three highest level of agreement were found between BWA and Minimap2, BBmap and Segemehl, Bowtie2 and Segemehl (Kappa 0.71, 0.74 and 0.77, respectively), while the three lowest level of agreement were found between Bowtie2 and Minimap2, Bowtie2 and BWA, Segemehl and Minimap2 (Kappa 0.05, 0.07 and 0.13, respectively). In addition, each aligner showed a portion of protein-encoding associated sequences within the multimapped reads, with the lowest number of distinct multimapped protein-encoding associated sequences found in BWA, followed by increasing numbers in Minimap2, BBmap, Bowtie2 and the highest in Segemehl;
2. All aligners showed differences in the assignment of reads to tRNA features, both in the number of specific tRNA genomic regions to which they assigned reads and in the different genomic copies of the same tRNAs (Table 5; Supplementary Table S1). When detecting tRNA-derived sRNAs, the specificity of the aligners decreased, and the sensitivity increased in the order BBmap-Minimap2-Bowtie2-BWA-Segemehl. The three highest level of agreement were found between BWA and Bowtie2, Minimap2 and BBmap, Minimap 2 and Bowtie2 (Kappa 0.61, 0.74 and 0.74, respectively), while the lowest level of agreement was found between Segemehl and any of four other aligners (Kappa 0). In addition, a subset of tRNAs was detected among the multimapped reads in the six samples by each aligner, with the lowest number of different multimapped tRNAs in BWA and a similarly low number in Bowtie2 and BBmap (their order varied depending on the sample), a higher number in Minimap2, and the highest in Segemehl;
3. While BWA, Bowtie2, Minimap2 and Segemehl assigned the same 37 rRNA-derived sRNAs in each sample (34 from chromosome I and three from chromosome II) and differed only in the number of reads assigned to each unique rRNA feature, BBmap assigned a smaller number of rRNA-derived sRNAs, ranging from 31 to 34 depending on the sample (28 to 31 from chromosome I and three from chromosome II). The reads that were not assigned to the rRNA feature by BBmap were all from chromosome I and were assigned to the following three to six (depending on the sample) genomic regions by four other aligners: VF_R0013, VF_R0022, VF_R0025, VF_R0026, VF_R0029 and VF_R0032, all of which were labeled as 5S RNAs (out of a total of 12 5S rRNAs in chromosome I); in addition, all rRNAs assigned by each aligner in each sample were detected among the multimapped reads.

**Table 4:** Overview of the sensitivity (Sn), specificity (Sp), and Kappa agreement differences between pairs of aligners for the unique mRNA-derived sRNAs in OMV from *A. fischeri*.

Aligner	Parameter	BBmap	Bowtie2	BWA	Minimap2	Segemehl
BBmap	Sn %	-	96.30 (95.50–98.0)	100	100	98.80 (97.40–99.40)
	Sp %	-	100	14.90 (5.10–28.60)	11.85 (4.80–27.30)	100
	Kappa	-	0.49 (0.44–0.58)	0.25 (0.09–0.44)	0.20 (0.09–0.42)	0.74 (0.50–0.89)
Bowtie2	Sn %	100	-	100	100	100
	Sp %	33.00 (29.40–39.50)	-	3.70 (3.10–7.90)	3.15 (1.20–3.90)	64.05 (54.50–77.10)
	Kappa	0.49 (0.44–0.58)	-	0.07 (0.06–0.18)	0.05 (0.02–0.07)	0.77 (0.69–0.87)
BWA	Sn %	98.05 (97.20–98.80)	94.10 (92.90–96.80)	-	100	96.05 (95.00–97.50)
	Sp %	100	100	-	55.60 (26.70–81.80)	100
	Kappa	0.25 (0.09–0.44)	0.07 (0.06–0.18)	-	0.71 (0.42–0.90)	0.16 (0.15–0.25)
Minimap2	Sn %	97.95 (96.70–98.70)	93.70 (92.70–96.80)	99.80 (99.50–99.90)	-	95.80 (94.90–97.50)
	Sp %	100	100	100	-	100
	Kappa	0.20 (0.09–0.42)	0.05 (0.02–0.07)	0.71 (0.42–0.90)	-	0.13 (0.12–0.26)
Segemehl	Sn %	100	98.00 (97.30–99.20)	100	100	-
	Sp %	60.70 (34.10–80.80)	100	9.35 (8.70–14.90)	7.40 (6.50–15.60)	-
	Kappa	0.74 (0.50–0.89)	0.77 (0.69–0.87)	0.16 (0.15–0.25)	0.13 (0.12–0.26)	-

The Sn, Sp, and Kappa values are presented as median with minimum and maximum values.

**Table 5:** Overview of the sensitivity (Sn), specificity (Sp), and Kappa agreement differences between pairs of aligners for the unique tRNA-derived sRNAs in OMV from *A. fischeri*.

Aligner	Parameter	BBmap	Bowtie2	BWA	Minimap2	Segemehl
BBmap	Sn %	-	93.50 (90.80–95.90)	90.35 (85.10–92.40)	96.20 (87.10–100)	82.55 (72.50–90.10)
	Sp %	-	100	100	100	100
	Kappa	-	0.53 (0.00–0.72)	0.10 (0.00–0.68)	0.74 (0.37–1.00)	0
Bowtie2	Sn %	100	-	96.40 (92.70–100)	100	90.45 (77.50–93.80)
	Sp %	40.00 (5.90–60.00)	-	100	61.90 (50.00–80.00)	100
	Kappa	0.53 (0.00–0.72)	-	0.61 (0.00–1.00)	0.74 (0.64–0.88)	0.00 (0.00–0.22)
BWA	Sn %	100	100	-	100	92.35 (80.00–97.50)
	Sp %	13.70 (7.60–57.10)	46.45 (2.90–100)	-	31.10 (16.70–75.00)	100
	Kappa	0.10 (0.00–0.68)	0.61 (0.00–1.00)	-	0.44 (0.26–0.84)	0.00 (0.00–0.47)
Minimap2	Sn %	100	95.45 (92.70–98.70)	95.25 (87.50–98.70)	-	87.00 (78.80–92.60)
	Sp %	61.90 (25.00–100)	100	100	-	100
	Kappa	0.74 (0.37–1.00)	0.74 (0.64–0.88)	0.44 (0.26–0.84)	-	0.00 (0.00–0.37)
Segemehl	Sn %	100	100	100	100	-
	Sp %	17.45 (9.90–27.50)	10.70 (6.20–22.50)	9.60 (2.50–33.30)	13.75 (7.40–25.00)	-
	Kappa	0	0.00 (0.00–0.22)	0.00 (0.00–0.47)	0.00 (0.00–0.37)	-

The Sn, Sp, and Kappa values are presented as median with minimum and maximum values.

**Figure 3:**
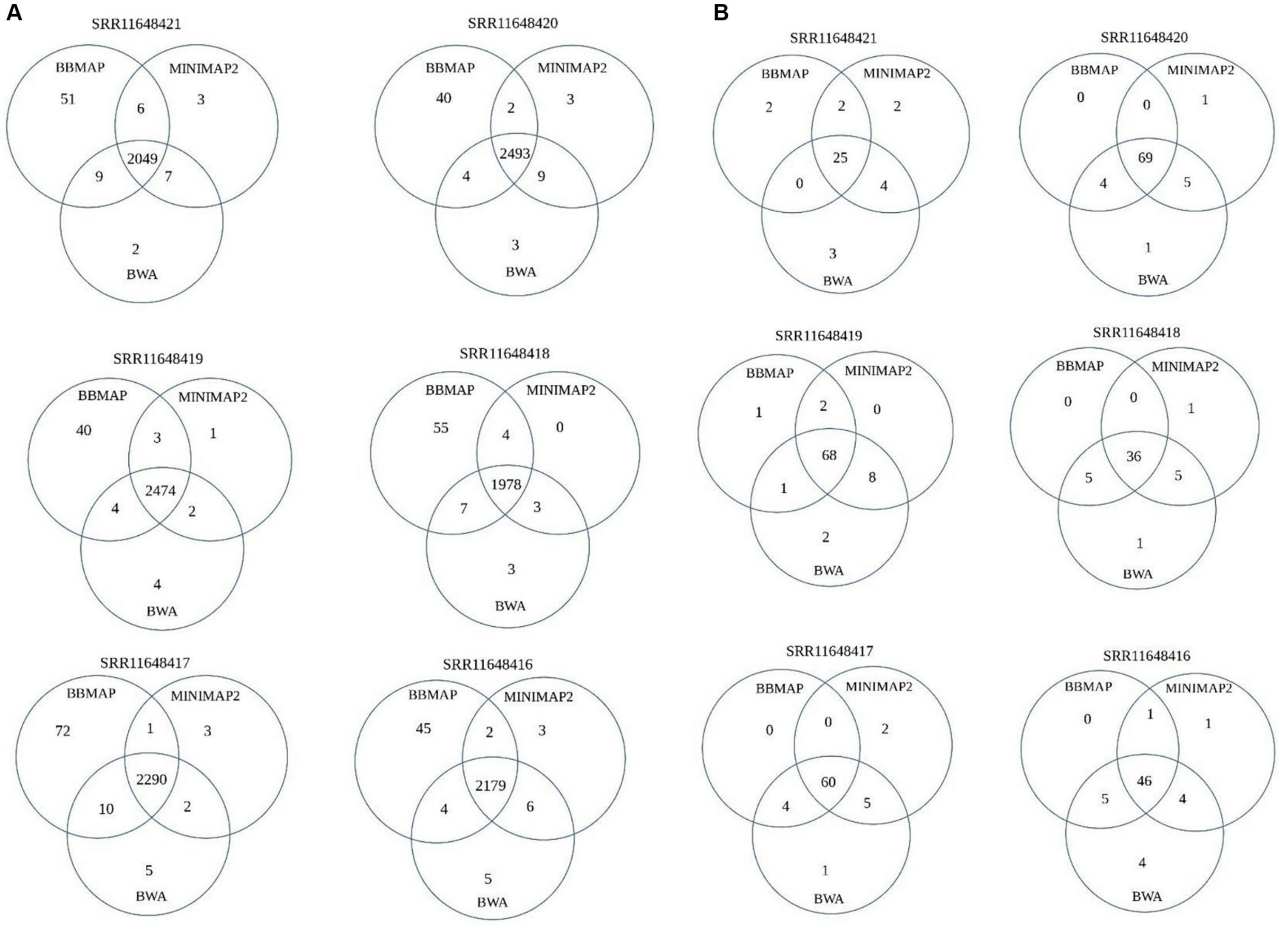
Venn diagram depicting the contribution of the intersect-then-combine approach with three aligners: BBmap, BWA and Minimap2. The diagram represents the number of unique mRNA-derived sRNAs **(A)** and tRNA-derived sRNAs **(B)** detected by all three aligners, by a pair of aligners and by a single aligner.

Given the significant differences in the performance of the five aligners, the intersect-then-combine approach was used with the three aligners that showed the best, yet different, overall performance as well as good combining ability – BBmap, BWA and Minimap2 to increase sensitivity and reduce false-positive detection of OMV sRNAs. This approach enabled the detection of 9–22 mRNA-derived, 6–11 tRNA-derived and 3–6 rRNA-derived sRNAs (depending on the sample) in the analyzed *A. fischeri* dataset, that would have otherwise been lost (all or some of them) (Figure 3; Supplementary Table S1).

## 4 Discussion

Although various benchmarking of different aligners have been performed on unrelated datasets to date, the question of aligner choice remains open because the correct alignment by each aligner depends on its algorithm and matrix, but is also affected by read size, reference genome size and repeats distribution and because the effects of reference genome properties have not yet been sufficiently explored (Phan et al., 2015; Xin et al., 2016; Song et al., 2023). Given the availability and development of numerous algorithms and tools for extracting information from different RNAseq data and the fact that each of them is the best choice under certain conditions, selecting the right aligner for a given dataset becomes a very important task. Most studies so far have been performed on DNAseq and RNAseq data, both long and short reads, mainly on eukaryotic genomes (Hatem et al., 2013; Baruzzo et al., 2017). Only to some extent have such studies been performed on prokaryotic genomes, mostly involving DNAseq- and metagenomics-related studies or various RNAseq-related tools considered individually as they were developed/tested (Gaur and Chaturvedi, 2017; Thankaswamy-Kosalai et al., 2017). To the authors’ knowledge none of the studies addressed the benchmarking of different aligners for the alignment of small RNA associated with bacterial OMVs to the bacterial reference genome. In this work, we tested five aligners known for their generally good performance, to map a specific set of small RNAs, associated with *A. fischeri* OMVs, to the reference genome. Each aligner was used with its default parameters. In addition to the basic metrics, we also tracked how the alignment generated by each aligner affected the recognition of the biotype of the OMV sRNAs.

Looking at the overall results of all aligners, BBmap showed the best basic metrics when considering alignment and assignment rates and one of the two fastest computational times. However, its’ MAPQ was the third best, it showed the third highest number of homopolimeric indels and its general error rate was the highest. In addition, BBmap assigned more reads to chromosome I than to chromosome II compared to all other aligners. Bowtie2 showed the second and third best basic metrics, also its MAPQ was the fourth best and it required more computational time – the fourth best compared to the others. Its mean coverage per chromosome II was statistically significantly higher in comparison to Segemehl and BBmap. On the other hand, BWA showed the third best basic metric, the highest percentage of homopolimeric indels, but also the best mean MAPQ, a short computational time (the second best) and the mean coverage of the chromosome II was as high as for Bowtie2. Minimap2 showed the fourth best basic metric, but also one of the two fastest computational times, the second best mean MAPQ and the third highest number of homopolimeric indels, as well as the same high mean coverage of chromosome II as Bowtie2 and BWA. Segemehl required the longest computational time and achieved the lowest mean MAPQ. It also had the lowest number of homopolimeric indels and while it had the second best assignment rate, it also had the lowest assignment rate. Its mean coverage of chromosome II was significantly lower than that of Bowtie2, BWA and Minimap2, but also significantly higher than that of BBmap.

The observed differences in alignment and assignment rates, coverage, mean MAPQ, mismatches and indels between the five aligners were to be expected considering that each alignment algorithm has its’ specific MAPQ ranges as well as rewards and penalties for matches and mismatches depending on the scoring matrix used. Since each aligner has specific advantages and disadvantages, these must be taken into account depending on the characteristics of the input dataset. A similar and expected conclusion was reached in another report (Donato et al., 2021), in which 17 aligners, including the five aligners we are interested in, were tested for their performance on different empirical and/or simulated human and mouse DNAseq and RNAseq datasets. The authors reported that each tool performed best under certain conditions and that the accuracy of the aligners varied for different RNA-Seq data. The overall scores assigned by the authors for empirical RNAseq data analysis performance for five aligners we are interested in were (in decreasing order): the highest for Segemehl and BWA-MEM, followed by BBmap and Minimap2, and finally Bowtie2. Since the organisms from which the datasets and reference genomes were derived were eukaryotic and of different length and complexity than the ones we tested, extrapolation would not be very trustworthy. However, since we lack benchmarking information for different aligners used to map different sRNA-seq reads on bacterial reference genomes, we could only refer to these data, although we are aware of all the limitations of such extrapolation.

While the issue of multimapping has been independently addressed on several occasions (Johnson et al., 2016; Bermúdez-Barrientos et al., 2020) unmapped and multimapped reads are usually not analyzed in detail in benchmark studies, but only their overall percentage is considered. In our study, the overall percentages of unmapped and multimapped reads were similar for each aligner, with a generally low percentage of unmapped reads and a high percentage of multimapped reads across all five aligners. In both categories, there were overrepresented sequences and kmers associated with each aligner, consistent with differences in the algorithms and scoring matrices of each aligner. Nevertheless, all five aligners shared a certain portion of overrepresented sequences and kmers in the unmapped and multimapped reads of the six samples. Among these overrepresented sequences there were no obvious patterns in terms of homopolymers or dinucleotide or trinucleotide repeats.

As expected, these alignment differences influenced the assignment of biotypes to OMV sRNAs. The assignment of reads to rRNA features was ambiguous/multiple for each aligner (in the numbers reported in the biotype-related results section), with all aligners giving the same final result when assigning multimapped reads to rRNA features except BBmap, which did not assign 3–6 5S rRNA-derived sRNAs in six samples compared to the other aligners. In contrast to the repetitive 5S rRNA genomic regions, the assignment of reads to tRNA features, which are as repetitive as rRNAs in the *A. fischeri* genome, was performed differently by each aligner. They agreed to some extent, but again, BBmap did not assign as many tRNA features as the other aligners. While BWA and Bowtie2 had an equally low number of multimapped tRNAs features as BBmap, Minimap2 assigned a higher number and Segemehl assigned the highest number across all six samples. The recognition of different tRNA-derived sRNAs was affected by these differences in each aligner (Supplementary Table S1), i.e., a certain part of reads in the six samples was assigned to the genomic feature tRNA-Phe (VF_T0013) by all aligners except BBmap, which assigned these reads to the other copies of tRNA-Phe, but not to this specific one. However, the greatest differences were observed in the assignment of reads to protein-encoding features (which have no copies in the *A. fischeri* genome). The lowest number of multimapped protein-encoding features was found in BWA (which ranked fourth in the number of distinct mRNA-derived sRNAs) and the highest in Segemehl (which ranked second in the number of distinct mRNA-derived sRNAs).

Considering all these results for the overall evaluation of the five aligners with their default settings, Segemehl should be avoided for this type of analysis, as it has the longest computational time, the highest CPU usage (data not shown) and the highest multimapping, especially with respect to tRNA and protein-coding features. Also, it showed extremely low agreement level for tRNAs with any of four other aligners (Kappa 0). The remaining four aligners showed overall good metrics and the pros and cons based on their overall performance were not entirely decisive in either case when tested with the *A. fischeri* genome and this particular OMV sRNAs dataset. Therefore, in this case it would be necessary to intersect the results of at least two (preferably three) differently performing aligners in order to extract the overlapping result for the downstream analysis. However, the differences obtained should not be discarded lightly, but should be carefully considered in the biological context of the dataset and the experiment as a whole, suggesting that the intersect-then-combine approach is the best choice for such analysis.

Of the remaining four aligners, Bowtie2 had the longest computational time, its MAPQ was the lowest and it showed extremely low agreement level with BWA and Minimap2 (Kappa <0.1 for both), which is why we excluded it. Considering all parameters, of the remaining three aligners, BBmap performed differently from the other two: BWA and Minimap2, which performed more similarly to each other than to BBmap. If we were to select more different pairs, BBmap should be intersected with one of these two other aligners. Since MAPQ is not always the best criterion (Wilton and Szalay, 2022) and a higher general error rate may be acceptable given the high editability of sRNAs, considering the number of individual biotypes assigned by each of the three aligners (as it is lower for BBmap than for the other aligners) and the number of multimapped reads per biotype, we would recommend intersecting BBmap (short computing time, lower rRNA and tRNA biotypes, medium number of pairwise differences in reads assigned/multimapped to protein-encoding, tRNA and rRNA features) with Minimap2 (short computing time, larger number of identified biotypes, lower number of pairwise differences in reads assigned/multimapped to protein-encoding, tRNA and rRNA features, more balanced coverage of chromosome II than by BBmap). If we had to select more similar aligners to improve the detection of overlapping alignments, we would recommend BWA and Minimap2 as they are more balanced compared to each other. However, since we are interested in increasing the overall sensitivity of OMV sRNAs detection, while reducing the number of falsely detected OMV sRNAs, we recommend using the intersection of Minimap2, BWA and BBmap and then combining the obtained results as (Minimap2⋂BWA + Minimap2⋂BBmap) or even better (Minimap2⋂BWA + Minimap2⋂BBmap+ BWA⋂BBmap), in order to reduce the loss of recognition of existing, biologically important OMV-associated sRNAs.

In summary, all five aligners tested in this particular analysis had specific advantages and disadvantages that need to be considered in future studies depending on the characteristics of the input OMV sRNAs dataset and the bacterial reference genome. Until we learn more about the effects of the dataset and reference genome on aligner performance, we recommend overlapping the results of at least two, preferably three, aligners to analyse bacterial OMV-associated sRNAs. Using the intersect-then-combine approach with three aligners would increase sensitivity and reduce the number of false positives, thereby improving the recognition of different sRNA biotypes. Given the use of the intersect-then-combine approach, false positives can be defined as alignments supported by only one aligner, and in this particular case the number of sRNAs detected by only one aligner. For example, as shown in Figure 3, this approach could exclude 45–80 false-positive mRNA-derived sRNAs and 2–7 false-positive tRNA-derived sRNAs from downstream analysis. For bacterial reference genomes with two circular chromosomes of different lengths and one circular plasmid, characterized by specific repetitive structures and containing copies of sequences with rRNA- and tRNA-related features and no copies of sequences with protein-encoding features (similar to *A. fischeri* reference genome) and for OMV-associated sRNAs datasets derived from such genomes, when using aligners with their default settings, we recommend avoiding Segemehl and using the intersect-then-combine approach with Minimap2, BWA and BBmap, to improve the identification of both overlapping and differently processed sRNA data for downstream analysis. Since we tested all these aligners with their default parameters, the results should improve even further if you tune the parameters of the selected aligners to the dataset (i.e., for BBmap reduce the allowed mismatches number, for Segemehl adjust the parameters for the allowed differences and the E-value, etc.). There are already many papers dealing with changing the default parameters of the five aligners, which can be helpful in narrowing down the options for testing with a particular dataset. However, even after adjusting the parameters for each tool to the analyzed dataset, taking into account the differences in their algorithms, the intersect-then-combine approach is still recommended, as it improves the sensitivity and specificity of sRNA detection. Nevertheless, in this paper, we intentionally tested the tools only with their basic default parameters, as this is often the case when biologists analyse NGS data. When looking at default settings of the aligners, besides the differences in performing global or local alignments, the number of allowed mismatches and the different penalization of mismatches/gaps, the default insert size of pair-end reads and/or intron size as well as the number of retained primary and secondary alignments were probably the main differences between the five aligners that could affect their results for the tested dataset.

Although the functions of the above-mentioned biotypes of sRNAs in *A. fischeri* are still unknown (except for some sRNAs from the ‘other’ class, which are reported to be essential for bioluminescent symbiosis with the squid *Euprymna scolopes,* carbohydrate metabolism, quorum regulation, etc.), general knowledge about the functions of mRNA-, tRNA- and rRNA-derived sRNAs differentially packaged in bacterial OMVs is growing. There is evidence that the composition of sRNAs in OMVs of *Pseudomonas aeruginosa* changes when exposed to external stress, including tRNA-Met-derived sRNA, which is involved in reducing the host immune response (Koeppen et al., 2016) or that Ile-tRF-5X in OMVs of *Escherichia coli* regulates host cell gene expression and proliferation (Diallo et al., 2022). Considering that these and other similar reports provide only a glimpse of the much broader functions of sRNAs that remain to be discovered, and that the composition of sRNAs packaged in OMVs can vary depending on the environment/interactions, the biological importance of distinct sRNA biotypes becomes clear, emphasizing the need to be able to detect them all. Since the detection of OMV-associated sRNAs depends on their alignment to the reference genome, given the variety of available aligners, it is important not to choose the aligner arbitrarily, but to consider which aligner or combination of aligners is best suited for the dataset/reference genome in question based on its performance results. Such an approach would help to improve reference genome alignment-based detection of existing, biologically important OMV-associated sRNAs.

## Data availability statement

The datasets presented have been deposited to the NCBI SRA database with accession number PRJNA12986 (https://www.ncbi.nlm.nih.gov/bioproject/PRJNA12986/).

## Author contributions

BB: Writing – review & editing, Writing – original draft, Visualization, Validation, Software, Investigation, Formal analysis, Data curation, Conceptualization. SN: Writing – review & editing, Validation, Formal analysis. IV: Formal Analysis, Visualization, Writing – review & editing. JS: Writing – review & editing, Resources. DN: Writing – review & editing, Supervision, Project administration, Funding acquisition.

## Footnotes

^1^ https://www.ncbi.nlm.nih.gov/bioproject/PRJNA629425

^2^ https://www.ncbi.nlm.nih.gov/bioproject/PRJNA12986/

## References

[ref1] Ahmadi Badi S. Bruno S. P. Moshiri A. Tarashi S. Siadat S. D. Masotti A. (2020). Small RNAs in outer membrane vesicles and their function in host-microbe interactions. Front. Microbiol. 11:1209. doi: 10.3389/fmicb.2020.01209, PMID: 32670219 PMC7327240

[ref2] Baldrich P. Rutter B. D. Karimi H. Z. Podicheti R. Meyers B. C. Innes R. W. (2019). Plant extracellular vesicles contain diverse small RNA species and are enriched in 10-to 17-nucleotide “tiny” RNAs. Plant Cell 31, 315–324. doi: 10.1105/tpc.18.00872, PMID: 30705133 PMC6447009

[ref3] Barik A. Das S. (2018). A comparative study of sequence-and structure-based features of small RNAs and other RNAs of bacteria. RNA Biol. 15, 95–103. doi: 10.1080/15476286.2017.1387709, PMID: 29099311 PMC5785981

[ref4] Baruzzo G. Hayer K. E. Kim E. J. Di Camillo B. Fitz Gerald G. A. Grant G. R. (2017). Simulation-based comprehensive benchmarking of RNA-seq aligners. Nat. Methods 14, 135–139. doi: 10.1038/nmeth.4106, PMID: 27941783 PMC5792058

[ref5] Bermúdez-Barrientos J. R. Ramírez-Sánchez O. Chow F. W. Buck A. H. Abreu-Goodger C. (2020). Disentangling sRNA-Seq data to study RNA communication between species. Nucleic Acids Res. 48:e21. doi: 10.1093/nar/gkz1198, PMID: 31879784 PMC7038986

[ref6] Bezuglov V. Stupnikov A. Skakov I. Shtratnikova V. Pilsner J. R. Suvorov A.. (2023). Approaches for sRNA analysis of human RNA-Seq data: comparison, benchmarking. Int. J. Mol. Sci. 24:4195. doi: 10.3390/ijms24044195 36835604 PMC9959513

[ref7] Bloch S. Węgrzyn A. Węgrzyn G. Nejman-Faleńczyk B. (2017). Small and smaller-sRNAs and MicroRNAs in the regulation of toxin gene expression in prokaryotic cells: a mini-review. Toxins 9:181. doi: 10.3390/toxins9060181, PMID: 28556797 PMC5488031

[ref8] Brantl S. Müller P. (2021). Cis-and trans-encoded small regulatory RNAs in *bacillus subtilis*. Microorganisms 9:1865. doi: 10.3390/microorganisms9091865, PMID: 34576762 PMC8464778

[ref9] Bushnell B. Rood J. Singer E. (2017). BBMerge–accurate paired shotgun read merging via overlap. PLoS One 12:e0185056. doi: 10.1371/journal.pone.0185056, PMID: 29073143 PMC5657622

[ref10] Caruana J. C. Walper S. A. (2020). Bacterial membrane vesicles as mediators of microbe-microbe and microbe-host community interactions. Front. Microbiol. 11:432. doi: 10.3389/fmicb.2020.00432, PMID: 32265873 PMC7105600

[ref11] Dauros-Singorenko P. Blenkiron C. Phillips A. Swift S. (2018). The functional RNA cargo of bacterial membrane vesicles. FEMS Microbiol. Lett. 365:fny023. doi: 10.1093/femsle/fny023, PMID: 29390056

[ref12] Diallo I. Ho J. Lalaouna D. Massé E. Provost P. (2022). RNA sequencing unveils very small RNAs with potential regulatory functions in bacteria. Front. Mol. Biosci. 9:914991. doi: 10.3389/fmolb.2022.914991, PMID: 35720117 PMC9203972

[ref13] Diallo I. Provost P. (2020). RNA-sequencing analyses of small bacterial RNAs and their emergence as virulence factors in host-pathogen interactions. Int. J. Mol. Sci. 21:1627. doi: 10.3390/ijms21051627 32120885 PMC7084465

[ref14] Donato L. Scimone C. Rinaldi C. D'Angelo R. Sidoti A. (2021). New evaluation methods of read mapping by 17 aligners on simulated and empirical NGS data: an updated comparison of DNA- and RNA-Seq data from Illumina and ion torrent technologies. Neural Comput. & Applic. 33, 15669–15692. doi: 10.1007/s00521-021-06188-z, PMID: 34155424 PMC8208613

[ref15] Felden B. Augagneur Y. (2021). Diversity and versatility in small RNA-mediated regulation in bacterial pathogens. Front. Microbiol. 12:719977. doi: 10.3389/fmicb.2021.719977, PMID: 34447363 PMC8383071

[ref16] Felden B. Gilot D. (2018). Modulation of bacterial sRNAs activity by epigenetic modifications: inputs from the eukaryotic miRNAs. Genes 10:22. doi: 10.3390/genes10010022, PMID: 30602712 PMC6356536

[ref17] Gaur P. Chaturvedi A. (2017). “A survey of bioinformatics-based tools in RNA-sequencing (RNA-seq) data analysis” in Translational bioinformatics and its application. eds. Wei D. Q. Ma Y. Cho W. Xu Q. Zhou F. (Dordrecht: Springer), 223–248.

[ref18] Goodhead I. Blow F. Brownridge P. Hughes M. Kenny J. Krishna R.. (2020). Large-scale and significant expression from pseudogenes in *Sodalis glossinidius* – a facultative bacterial endosymbiont. Microb Genom 6:e000285. doi: 10.1099/mgen.0.000285, PMID: 31922467 PMC7067036

[ref19] Hatem A. Bozdağ D. Toland A. E. Çatalyürek Ü. V. (2013). Benchmarking short sequence mapping tools. BMC Bioinformatics 14, 1–25. doi: 10.1186/1471-2105-14-184, PMID: 23758764 PMC3694458

[ref20] Hoffmann S. Otto C. Kurtz S. Sharma C. M. Khaitovich P. Vogel J.. (2009). Fast mapping of short sequences with mismatches, insertions and deletions using index structures. PLoS Comput. Biol. 5:e1000502. doi: 10.1371/journal.pcbi.1000502, PMID: 19750212 PMC2730575

[ref21] Iosub I. A. Marchioretto M. van Nues R. W. McKellar S. Viero G. Granneman S. (2021). The mRNA derived MalH sRNA contributes to alternative carbon source utilization by tuning maltoporin expression in *E. coli*. RNA Biol. 18, 914–931. doi: 10.1080/15476286.2020.1827784, PMID: 33043783 PMC8081044

[ref22] Johnson N. R. Yeoh J. M. Coruh C. Axtell M. J. (2016). Improved placement of multi-mapping small RNAs. G3 6, 2103–2111. doi: 10.1534/g3.116.030452, PMID: 27175019 PMC4938663

[ref23] Koeppen K. Hampton T. H. Jarek M. Scharfe M. Gerber S. A. Mielcarz D. W.. (2016). A novel mechanism of host-pathogen interaction through sRNA in bacterial outer membrane vesicles. PLoS Pathog. 12:e1005672. doi: 10.1371/journal.ppat.1005672, PMID: 27295279 PMC4905634

[ref24] Langmead B. Salzberg S. (2012). Fast gapped-read alignment with bowtie 2. Nat. Methods 9, 357–359. doi: 10.1038/nmeth.1923, PMID: 22388286 PMC3322381

[ref25] Langmead B. Trapnell C. Pop M. Salzberg S. L. (2009). Ultrafast and memory-efficient alignment of short DNA sequences to the human genome. Genome Biol. 10, R25–R10. doi: 10.1186/gb-2009-10-3-r25, PMID: 19261174 PMC2690996

[ref26] Li H. (2018). Minimap2: pairwise alignment for nucleotide sequences. Bioinformatics 34, 3094–3100. doi: 10.1093/bioinformatics/bty191, PMID: 29750242 PMC6137996

[ref27] Li H. Durbin R. (2009). Fast and accurate short read alignment with burrows-wheeler transform. Bioinformatics 25, 1754–1760. doi: 10.1093/bioinformatics/btp324, PMID: 19451168 PMC2705234

[ref28] Li Z. Stanton B. A. (2021). Transfer RNA-derived fragments, the underappreciated regulatory small RNAs in microbial pathogenesis. Front. Microbiol. 12:687632. doi: 10.3389/fmicb.2021.687632, PMID: 34079534 PMC8166272

[ref29] Moriano-Gutierrez S. Bongrand C. Essock-Burns T. Wu L. McFall-Ngai M. J. Ruby E. G. (2020). The noncoding small RNA SsrA is released by Vibrio fischeri and modulates critical host responses. PLoS Biol. 18:e3000934. doi: 10.1371/journal.pbio.3000934, PMID: 33141816 PMC7665748

[ref30] Musich R. Cadle-Davidson L. Osier M. V. (2021). Comparison of short-read sequence aligners indicates strengths and weaknesses for biologists to consider. Front. Plant Sci. 12:657240. doi: 10.3389/fpls.2021.657240, PMID: 33936141 PMC8087178

[ref31] Phan V. Gao S. Tran Q. Vo N. S. (2015). How genome complexity can explain the difficulty of aligning reads to genomes. BMC Bioinformatics 16, 1–15. doi: 10.1186/1471-2105-16-S17-S3, PMID: 26678826 PMC4674900

[ref32] Ponath F. Hör J. Vogel J. (2022). An overview of gene regulation in bacteria by small RNAs derived from mRNA 3′ ends. FEMS Microbiol. Rev. 46:fuac017. doi: 10.1093/femsre/fuac017, PMID: 35388892 PMC9438474

[ref33] Raabe C. A. Tang T. H. Brosius J. Rozhdestvensky T. S. (2014). Biases in small RNA deep sequencing data. Nucleic Acids Res. 42, 1414–1426. doi: 10.1093/nar/gkt1021, PMID: 24198247 PMC3919602

[ref34] Ren B. Wang X. Duan J. Ma J. (2019). Rhizobial tRNA-derived small RNAs are signal molecules regulating plant nodulation. Science 365, 919–922. doi: 10.1126/science.aav8907, PMID: 31346137

[ref35] Sartorio M. G. Pardue E. J. Feldman M. F. Haurat M. F. (2021). Bacterial outer membrane vesicles: from discovery to applications. Ann. Rev. Microbiol. 75, 609–630. doi: 10.1146/annurev-micro-052821-031444, PMID: 34351789 PMC8500939

[ref36] Song B. Buckler E. S. Stitzer M. C. (2023). New whole-genome alignment tools are needed for tapping into plant diversity. Trends Plan Sci 29, 355–369. doi: 10.1016/j.tplants.2023.08.013, PMID: 37749022

[ref37] Sousa J. P. Silva A. F. Q. Arraiano C. M. Andrade J. M. (2023). “Bacterial Small RNAs: Diversity of Structure and Function” in RNA Structure and Function. RNA Technologies. ed. Barciszewski J. (Cham: Springer), 259–277.

[ref38] Tepavčević J. Yarrington K. Fung B. Lin X. Visick K. L. (2022). sRNA chaperone Hfq controls bioluminescence and other phenotypes through Qrr1-dependent and -independent mechanisms in *Vibrio fischeri*. Gene 809:146048. doi: 10.1016/j.gene.2021.146048, PMID: 34756963 PMC8673744

[ref39] Thankaswamy-Kosalai S. Sen P. Nookaew I. (2017). Evaluation and assessment of read-mapping by multiple next-generation sequencing aligners based on genome-wide characteristics. Genomics 109, 186–191. doi: 10.1016/j.ygeno.2017.03.001, PMID: 28286147

[ref40] Wilton R. Szalay A. S. (2022). Performance optimization in DNA short-read alignment. Bioinformatics 38, 2081–2087. doi: 10.1093/bioinformatics/btac066, PMID: 35139149 PMC10060706

[ref41] Xin H. Nahar S. Zhu R. Emmons J. Pekhimenko G. Kingsford C.. (2016). Optimal seed solver: optimizing seed selection in read mapping. Bioinformatics 32, 1632–1642. doi: 10.1093/bioinformatics/btv670, PMID: 26568624 PMC6363230

[ref42] Zwieb C. Wower I. K. Wower J. (1999). Comparative sequence analysis of tmRNA. Nucleic Acids Res. 27, 2063–2071. doi: 10.1093/nar/27.10.2063, PMID: 10219077 PMC148424

